# A Curriculum Design and Teaching Experience Created by and for Bioscience Postdoctoral Fellows in a Medical School

**DOI:** 10.1007/s40670-019-00889-w

**Published:** 2019-12-13

**Authors:** Esther Nuebel, Sara M Nowinski, Casey W Hemmis, Janet E Lindsley

**Affiliations:** 1 Department of Biochemistry, University of Utah School of Medicine, Salt Lake City, UT 84112 USA; 2 Howard Hughes Medical Institute, 4000 Jones Bridge Rd, Chevy Chase, MD 20815 USA

**Keywords:** Postdoctoral training, Curriculum design, Backwards design, Just-in-Time Teaching, Team-based learning, Ownership

## Abstract

Many medical school postdoctoral fellows (postdocs) lack training in curriculum design and student-centered instruction. A team of bioscience postdocs and a medical school curriculum assistant dean co-created an experience to fill this gap. Kern’s and Kirkpatrick’s frameworks were used for the design and evaluation, respectively, of both the postdoc experience and the undergraduate course they developed. Postdocs taught the course using student-centered methods, especially team-based learning and Just-in-Time Teaching. Following a successful pilot phase, this low resource postdoc experience and undergraduate course are regularly offered. Participating postdocs develop the knowledge, skills, and attitudes to effectively participate in medical school education.

## Background

A group of PhD postdoctoral fellows (postdocs, including authors CWH, SMN, and EN) in the Biochemistry Department at the University of Utah School of Medicine (UUSOM) identified a gap in their training: there was no way for them to gain meaningful experience in curriculum design and teaching while pursuing biomedical research. They recognized their need to build their educational skillset, in addition to acquiring scientific accolades in order to be competitive for, and ultimately successful in, faculty positions. While they had functioned as teaching assistants in graduate school, and there were local opportunities to participate in existing courses, none of these options provided an experience to create a new curriculum or develop skills in learner-centered instruction.

The postdocs’ objective to attain educator training aligns well with the need for faculty having these skill sets. Most medical schools invest significantly in faculty development initiatives for enhancing teaching effectiveness [1] and in sending faculty to educational conferences to learn these skills. Medical school administrators are grateful to work with faculty who already understand the basics of student-centered learning and instructional design (including author JEL).

In order to address both the needs of the postdocs and medical educators, we developed a new postdoc training experience based on frameworks of learner ownership [2] and collaborative learning [3]. Ownership is an implicit element of constructivist learning theory; educational programs based on ownership focus on the needs, interests, and experiences of the learner while giving them the responsibility, autonomy, and power over their outcomes [2]. Research mentors often describe successful graduate students and postdocs as “taking ownership of their projects.” Research labs also depend on collaborative learning, with lab members helping each other develop new skills and evaluating each other’s ideas. The foundations of this experience are therefore readily understood by all participants.

## Activity

In spring of 2018, we initiated a pilot postdoc training experience in curriculum design and student-centered teaching. The biology department at our university had identified a need for new summer senior-level courses and therefore welcomed our partnership. We utilized Kern’s model for curriculum design [4], with an emphasis on backwards design principles [5, 6], to create both the new postdoc training experience and a new undergraduate biology course designed and taught by the postdocs, summarized in Table 1. The postdocs therefore functioned alternately as learners, teachers, and co-creators [7, 8].

**Table 1:** Application of Kern’s curriculum design steps to both the postdoc training experience and the biology course creation

Kern’s curriculum design step	Postdoctoral training experience	Undergraduate biology course
1. Problem Identification and General Needs Assessment	1. Opportunities for curriculum design and utilizing goal-appropriate, student-centered teaching techniques	1. Teaching on how to read primary research literature 2. Learning to critically analyze and discuss primary literature research results and conclusions
2. Targeted Needs Assessment	1. Three UUSOM postdocs willing to work long hours to achieve their goal of learning to design a new course and teaching it 2. Lack of financial resources to support development of an extensive program 3. Desire of the University of Utah Biology department to offer new, 5000-level undergraduate courses 4. Availability of medical school assistant dean willing/able to serve as an education mentor 5. Willingness of postdoc research mentors to allow postdocs to take some time away from the lab bench to pursue additional academic training	3. Lack of summer senior-level Biology courses 4. Lack of process-focused courses to meet biology department Program Objectives
3. Goals and Objectives	1. Provide a mentored opportunity for postdocs to design and teach a course on a topic of their choosing related to biochemistry and cellular biology 2. Postdocs will be able to utilize the Kern’s model of curriculum design and backwards design principles to design an effective new course 3. Postdocs will be able to utilize modern, student-centered methods of teaching, as appropriate to achieve the goals and objectives of their course 4. Postdocs will be able to utilize Kirkpatrick’s framework for curriculum evaluation to analyze the effectiveness of their course	1. Read a scientific paper, be able to understand and summarize it, and have an opinion about it 2. Describe the different research topics that fall under the umbrella of biochemistry (structural, bioenergetics, metabolic pathways, signaling, more)^1^ 3. Explain specific, cutting-edge research that bridges a wide-range of topics in mitochondrial metabolism including: electron transport chain supercomplexes, ATP production and signaling, reactive oxygen species, metabolic control of cell growth, and metabolism in disease^1^ 4. Use scientific databases to search for research papers and review articles
4. Educational strategies	1. Small group discussion 2. Just-in-time Teaching 3. Multi-step mentor and peer feedback on design steps and teaching strategies	1. Team-based learning (TBL) 2. Just-in-time Teaching (JiTT) 3. Oral presentations
5. Implementation	1. Weekly meetings between postdocs and mentor to jointly determine process and deadlines 2. Mentor explained curriculum design principles (Kern’s and backwards design) and introduced student-centered teaching strategies 3. Postdocs drafted course goals and assessments, chose and sequenced primary literature research articles and session objectives; mentor reviewed and provided feedback 4. Postdocs aligned teaching techniques with session objectives, developed detailed lesson plans, developed the Learning Management System site; mentor provided feedback 5. Postdocs were the course instructors; mentor provided feedback on teaching and led critical reflection/debriefing sessions throughout the 5-week course	1. 2-credit BIOL 5960; 11 × 3 h sessions 2. Each of 3 postdocs was the primary instructor for 3 sessions, but all participated in all classes 3. Type class session: students completed pre-work before class and submitted questions used for JiTT. In class, students completed individual Readiness Assurance Test (iRAT), followed by group Readiness Assurance Test (gRAT). After completion of RATs, primary instructor responded to student questions (RATs and JiTT). Remainder of class time was spent on group application exercises (GAEs) designed to achieve learning objectives 4. One of the postdocs designed and maintained the course LMS site 5. Communication, including feedback and grades, was facilitated in near-real-time through LMS
6. Evaluation and Feedback	See Table 2 for application of Kirkpatrick framework	1. See Table 2 2. Peer feedback 3. Mentor feedback

^1^Goals specific for 2018 iteration of the course. In 2019 the new cohort of postdocs chose to focus on apoptosis and autophagy with an overlapping but distinct set of course goals

The postdocs and education mentor met weekly to design the curriculum. The emphasis of these meetings was always backwards design and careful alignment of all assessments, session objectives, materials, and teaching methods to course goals. The team set aggressive deadlines for drafting and finalizing design phases, with the education mentor providing extensive feedback initially and the postdocs providing increasing amounts of peer feedback during the process. Once the course goals and objectives were clearly defined, the final assessment was designed: the undergraduates would individually give an oral presentation about a new primary literature research paper and respond to instructor questions. This ambitious target provided clarity on what the course would need to accomplish, both in terms of content and process. Other primary literature articles were chosen to have specific sections slowly analyzed during the course in a scaffolded progression. Team-based learning (TBL) [9] and Just-in-Time Teaching (JiTT) [10] were chosen as the primary tools based on the published evidence of their effectiveness in helping students reach higher level cognitive skills [11, 12]. The mentor provided a mini-workshop on TBL, the template utilized for creating medical student TBLs at the UUSOM, and examples of JiTT. Session objectives were finalized; lesson plans, pre-session homework, and specific pre-session learning objectives were created. The learning management system (Canvas) site was designed for clarity and ease of use by students, with each session module set up identically. To help with JiTT, each pre-session assignment included prompts for students to submit questions about what confused them and what they found most interesting in the pre-work.

Following this intensive design phase, the postdocs taught the 5-week, 6-h per week, 2-credit course entitled *Understanding Peer-Reviewed Literature: Focus on Mitochondrial Metabolism*. Four senior Biology majors registered for the course. The education mentor observed an early class session and facilitated a discussion with the postdocs afterwards. After each session, the postdocs critically reflected about their experiences and identified potential ways to improve before the next session. Postdocs also provided peer feedback to each other after each session.

Kirkpatrick’s framework for curriculum evaluation was used to evaluate the success of both the postdoc training experience and their biology course (see Table 2). While no formal certificate is provided to postdocs completing this experience, the mentor provided detailed letters of evaluation for the postdocs.

**Table 2:** Application of the Kirkpatrick framework for learning evaluation

Evaluation level	Postdoc training experience	Results	Undergraduate biology student experience	Results
1. Reaction	1. Informal postdoc and education mentor reflections on perceived impact and satisfaction 2. Research mentor survey on impact and satisfaction	1. Informal perceptions of high impact and satisfaction 2. Informal perceptions of high impact and satisfaction	1. Quality of JiTT pre-session questions and points of interest submitted 2. University course evaluation survey 3. Biology department curriculum representative survey on satisfaction and program continuation	1. Informally judged to be thoughtful and engaged 2. Overall course evaluation score of 5.96/6.00, significantly above departmental average. The question with the largest positive differential was “This course has increased my capacity for analytical thinking and expression” 3. Both faculty and staff in the biology department have provided written statements of satisfaction and desire for program to continue
2. Learning	Informally assessed by education mentor based on postdocs’ demonstrated abilities to appropriately apply backwards design principles, create scaffolded learning experiences and align all session objectives, assessments, and materials to the course goals	The postdocs demonstrated skills in creating goals and mapping out an entire course, creating TBL sessions, writing assessments (multiple-choice, open ended and oral) and grading rubrics that match the learning objectives, and creating a supportive classroom culture	1. Student performance on readiness assurance tests and in-class learning activities 2. Student performance on the end-of-course oral assessment	1. Average iRAT scores for each class period ranged from 6 to 9 out of 10 with a mean of 7.25. gRAT scores for each class period ranged from 8.5–10 with a mean of 9.6. The mean score on the final assessment was 90%. Additionally, iRAT and gRAT difficulty increased significantly over the duration of the course, with no decrease in performance. Instructors noted insightfulness of student submitted pre-work questions and comments during GAEs also improved throughout the course 2. Students were largely able to interpret a new research article, make choices about which data were important to include or exclude, while maintaining the scientific narrative described in the publication. They were also able to provide alternative interpretations of the data in the paper, and successfully design experiments for hypothetical future research directions
3. Behavior	Did postdocs apply backwards design principles to other academic activities?	Two of the postdocs specifically identified using backwards design when developing research presentations since completion of this experience	Have the students shown evidence of increased interest in biomedical research?	One undergraduate student joined the biochemistry lab of the two of the postdocs after taking BIOL5960
4. Results	Evidence of impact of program on postdoc job offers	One of the postdocs received a faculty position offer from a prestigious college that specifically referenced his unique curriculum design and student-centered teaching experience. The other two postdocs have not yet entered the job market	NA	NA

## Results and Discussion

Based on the evaluation measures utilized (see Table 2), the postdocs successfully designed and taught their own course. The undergraduate students were engaged throughout and performed well on the final oral assessment and throughout the course (Table 2). The final course ratings by the undergraduate students were high, well above the departmental average. The postdocs felt that their learning needs were met and that the intensive and relatively short (about 4 months total) nature of the experience prevented excessive disruption to their biochemistry research. Several months after the experience ended, one of the postdocs received a faculty position offer from a prestigious college that specifically referenced his unique curriculum design and student-centered teaching experience.

The biology and biochemistry departments, as well as the education mentor, also found the experience to be successful. The initial pilot program is now an official training opportunity for bioscience postdocs at the UUSOM. The biology department curriculum committee approved a regular course to be offered each summer (BIOL 5800 – Advanced Topics in Biochemistry and Cell Biology: Developing Skills in Reading and Interpreting Primary Literature with a Focus on X). During the summer of 2019, two postdocs participated, designing and teaching a course to 9 undergraduates on apoptosis and autophagy.

In addition to the standard academic measures of success, the participants in this experience found it deeply satisfying. Just like in their scientific research projects, the success of this experience depended on the postdocs *owning* the biology course [2]. Their initial struggles with clearly defining the course goals and aligning all activities with these goals paid off in the joy of helping students achieve challenging expectations. The education mentor enjoyed the opportunity to work with a team of postdocs. This experience created a sense of community, based on both a shared purpose and the fun of working creatively together in a safe and supportive team. Given the prevalence of burnout and its relation to feeling isolated [13], this outcome should not be overlooked.

The need for postdoc training in education has been well documented [14], and several programs already exist to address this need [15–17]. The postdoc training experience described here differs from these other programs in several ways. (1) Our training experience is for postdocs in a medical school and is led by a medical school curriculum dean, while the postdocs teach an undergraduate biology course. Therefore, the postdocs are exposed to the perspectives, methodologies, and cultures of both medical school and undergraduate STEM teaching. (2) Our training experience is a short and intense work-based opportunity, with postdocs jumping right into curriculum design with no didactic preamble. (3) Other than the time invested by the postdocs (about 200–260 h) and the teaching mentor (about 30 h/year), there is no cost associated with this experience and the biology department gains a well-designed and effectively taught course at no cost. It should be possible to create similar training experiences at other medical schools that have bioscience postdocs with academic goals and undergraduate campuses located nearby. The limitations of the experience include its impact on a relatively small number of learners (to date five postdocs and 13 undergraduates over 2 years) and its implementation at only one US-based university and medical school.

In conclusion, we describe a low-resource, highly authentic postdoc curriculum design and teaching experience that utilizes design, evaluation frameworks, and teaching methods commonly used in medical education. These postdocs learned how much work goes on behind the scenes of an effective course, and should be well prepared for the educational portion of their academic careers.
